# Heartworm-associated respiratory disease (HARD) induced by immature adult *Dirofilaria immitis* in cats

**DOI:** 10.1186/s13071-017-2452-6

**Published:** 2017-11-09

**Authors:** A. Ray Dillon, Byron L. Blagburn, Michael Tillson, William Brawner, Betsy Welles, Calvin Johnson, Russell Cattley, Pat Rynders, Sharron Barney

**Affiliations:** 1 College of Veterinary Medicine, Auburn University, Auburn, Alabama 36849 USA; 2 Department of Clinical Sciences, College of Veterinary Medicine, Auburn University, 1220 Wire Road, Auburn, Alabama 36849 USA

**Keywords:** Feline, Heartworm, *Dirofilaria immitis*, Respiratory disease, HARD, Myofibrocyte

## Abstract

**Background:**

A controlled, blind research study was conducted to define the initial inflammatory response and lung damage associated with the death of immature adult *Dirofilaria immitis* in cats as compared with cats developing adult heartworm infections and cats on preventive medication.

**Methods:**

Three groups of cats were utilized, 10 per group. All cats were infected with 100 third-stage (L3) larvae by subcutaneous injection. Group A cats were treated topically with selamectin (Revolution®; Zoetis) per label directions at 28 days post infection (PI) and once monthly for 8 months. Group B cats were treated orally with ivermectin (Ivomec®; Merial) at 150 μg/kg at 70 days PI, then every 2 weeks for 5 months. Group C cats were untreated PI. At baseline (Day 0) and on Days 70, 110, 168, and 240 PI, peripheral blood, serum, bronchial lavage, and thoracic radiographic images were collected on all cats. Upon completion of the study (Day 245), cats were euthanized and necropsies were conducted.

**Results:**

Results were analyzed statistically between groups by ANOVA and by paired sample T testing for changes within the group over time. The selamectin-treated cats (Group A) did not develop radiographically evident changes throughout the study and were free of adult heartworms or worm fragments at necropsy. The heartworm life cycle was abbreviated with oral doses of ivermectin (Group B), shown by the absence of adult heartworms or worm fragments at necropsy. The early stage of immature adult worm in Group B cats, however, did induce severe pulmonary airway, interstitial, and arterial lung lesions, revealing that the abbreviated infection is a significant cause of respiratory pathology in cats. Cats in Groups B and C could not be differentiated based on radiographic changes, serologic antibody titers, complete blood count, or bronchoalveolar lavage cytology at any time point throughout the study. Eighty percent of cats in Group A and 100% of cats in Groups B and C became heartworm antibody positive at some time point post infection.

**Conclusions:**

The clinical implications of this study are that cats that become infected with immature adult heartworms may not develop fully mature heartworms and are only transiently heartworm antibody positive, but do develop Heartworm-Associated Respiratory Disease (HARD).

## Background

By common definition, *Dirofilaria immitis* is discussed as having a 6-month life cycle (infection of host through development and sexual maturity) [1–4]. The assumption that clinical disease does not develop until the parasite is a 6-month-old adult is not consistent with clinical experience [5, 6].

The initial arrival of immature L5 in the small pulmonary vessels of the lungs is associated with an intense eosinophilic pulmonary reaction and clinical and radiographic signs may be present in this 3- to 6-month post-infection period. [7, 8] This 3-month disease cycle precedes the production of microfilaria and circulating antigen by 2 to 3 months. Because of the difference in the host immune reaction [9] and higher mortality of the immature L5 worms in cats than dogs, (8) the clinical signs, diagnosis, and effects of prophylaxis are different in the cat compared with the dog with heartworm infection [1, 7, 10, 11].

After a mosquito acquires the microfilaria (L1), adequate exposure to warm temperatures must occur during the life span (1 month) of many of the mosquito vectors. The infective larvae are deposited on the skin of an animal when the mosquito feeds again and the L3 enters through the bite wound. A maximum of 10 to 12 L3 can be transmitted by a single mosquito. The L3 stages molt to L4 and L5 (adults) and migrate to the pulmonary arteries arriving as L5 approximately 70 to 90 days after infection. These small (1–2 cm in length) L5 are distributed mainly to the caudal distal pulmonary arteries, and by 6 to 7 months PI develop to sexually mature adults and migrate back toward the right ventricle [2, 3, 8, 10, 12]. If both sexes are present, microfilariae are produced 6 to 7 months after L3 infection and can be detected in the blood in the dog, but rarely in the cat. The common detection methods for adult antigen are positive typically about 6 to 7 months after infection. High enough quantities of the glycoprotein to be detected are only associated with fully mature adult female heartworms [1, 11, 13].

The purpose of this study was to determine, over the initial 8 months after infection, the response of the feline lung to the initial arrival and early death of young immature heartworms (HWs) that do not develop into adult HWs and to determine the effects of an abbreviated immature HW infection in cats 8 months after infection. Many veterinarians assume that when cats develop immature HWs in the pulmonary arteries and lungs, most cats will “self-cure” and the worms will die without any consequence to the cat [10, 12–14]. Research has demonstrated, however, that the early infection is associated with an intense inflammatory response [2, 8]. In addition, clinical experience and a prospective clinical study of cats with suspected HW disease has indicated that some cats will get the early infection and the worm(s) will die, but the cat will continue to have chronic inflammatory lung disease [15–17].

## Methods

To determine the initial inflammatory response associated with immature HW death, three groups (*n* = 10) of specific pathogen-free neutered male and spayed female 6-month-old cats were utilized (Groups A, B, and C). All cats were infected with 100 L3 larvae (Missouri strain) by subcutaneous (SQ) injection into the flank.

Group A cats served as controls for the absence of adult HWs. After infection, the cats were treated with topically applied selamectin (Revolution®, Zoetis), dosage based on body weight and weight range as indicated by label, once per month beginning 28 days PI to kill the immature larvae before they reach the pulmonary arteries.

Group B cats were infected and the larvae were allowed to mature for 70 days. At 70 days PI, ivermectin (Ivomec®, Merial), 150 μg/kg per os, was administered every 2 weeks for 5 months.

Group C cats served as the positive control for the study and were infected with L3 larvae and left untreated, thus allowing larvae to mature into adult HWs.

All groups of cats were housed as isolated groups in the indoor animal rooms of the Laboratory Animal Health Veterinary Research Building at Auburn University to prevent exposure to mosquitoes that might be harboring HW larvae. The protocol was approved by the Auburn University Institutional Animal Care and Use Committee and was conducted in an AAALAC-accredited, environmentally isolated facility.

Cats were observed for a period of 8 months PI. At baseline (Day 0) and on Days 70, 110, 168, and 240 PI, peripheral blood for complete blood count (CBC) and serum for serology was collected. On Days 0, 110, 168, and 240 bronchoalveolar lavage (BAL) with 10 mL of lactated Ringers solution was performed, and radiographic ventrodorsal (VD) and lateral thoracic images were acquired under sedation with an intramuscular (IM) dose of medetomidine (Domitor®, Zoetis), butorphanol (Torbugesic®, Zoetis), and ketamine (ketamine hydrochloride, Zoetis). Following the BAL and radiograph procedures an IM dose of atipamezole (Antisedan®, Zoetis) was administered. Cats were monitored daily and physical examinations performed weekly.

At the termination of the study (Day 245), cats were humanely euthanized under sedation using pentobarbital sodium and phenytoin sodium solution (Euthasol®, Virbac AH), 1 mL/10 lbs. given intraperitoneally (IP), and complete necropsies were conducted with collection of lung, heart, brain, kidney, and liver for histopathology studies. Immediately post mortem a blood sample from the right ventricle was collected for serology. Right caudal lung lobes were fixed perfused with 10% formalin via the bronchi to a pressure of 14 cm H_2_O. Pathologists and radiologists were blinded to the treatment groups to which cats were assigned, creating a controlled, blind study format.

Data reported in this paper include results from thoracic radiographs, complete peripheral blood counts, serology, BAL cytology, and lung histopathology.

Evaluation of thoracic radiographs consisted of severity scoring (0–3) for 12 different parameters, including bronchial, parenchymal, vascular, and cardiac changes. Histopathology of lung tissue stained with H & E and smooth muscle actin (HSRL, Inc) was based on subjective evaluation of severity (scoring 0–3) of pathology of the right caudal lung lobe, including pulmonary artery, pulmonary arterioles, bronchi, bronchioles, and alveolus including interstitial smooth muscle proliferation. For the bronchioles, the airways were measured for morphometry by calculating the cross-sectional area of the lumen, area of bronchial wall and lumen, and the area of the bronchial wall. A minimum of five bronchioles were measured on photographic digital images for each cat using ImageJ (NIH, http://rsb.info.nih.gov/ij/). Data were analyzed to compare the lumen to wall ratio, total area to wall ratio, and total area to lumen ratio. The BAL cytology data (Clinical Pathology Laboratory, Auburn University, College of Veterinary Medicine) were recorded as subjective descriptive narrative and expressed cellular morphology as percentage of cell types compared with nucleated cells observed. Serologic evaluation of batched frozen serum was performed spectrophotometrically (Antech Diagnostics) with DiroCHECK® (Symbiotics; currently Zoetis) for HW antigen and enzyme-linked immunosorbent assay (ELISA) for HW antibody.

All statistical analysis of data was performed with Systat Software (Sigma Plot 12, Systat Software Inc). Data points across time within each group were evaluated by paired T testing for changes between collection dates. Data between groups was evaluated with ANOVA differences between groups. Pearson’s and Spearman’s correlations were performed to evaluate linkage between histologic, radiographic, serologic, hematologic, and BAL results.

## Results

### Necropsy

No live HWs or HW fragments were found in any of the selamectin (Revolution®)-treated cats (Group A) at necropsy. Of the Group A cats, 80% were HW antibody (Ab) positive on at least one sampling interval during the study. None of the Group A cats were HW antigen (Ag) positive.

**Fig. 1:**
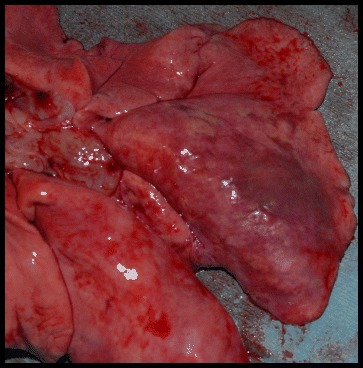
Lung of cat in Group B before pressure perfusion fixation. The lungs are turgid and especially the right and left caudal lung lobes will not deflate. Discoloration of surface can be visualized in the right caudal lung lobe. No adult HWs were present

One live immature adult HW (8 cm) and a 7-cm HW fragment were found in one of the Group B [abbreviated infection, ivermectin (Ivomec®) treated] cats. This cat was also HW Ag positive and was not considered to have an abbreviated HW infection; thus results were excluded from statistical evaluation. One Group B cat died of respiratory distress at 4 months PI. Upon necropsy, more than 10 immature dead HWs (3–4 cm) and numerous fragments were observed. The remaining eight cats in Group B were free from HWs or HW fragments at necropsy on Day 245, and were HW Ag negative. All of the Group B cats were HW Ab positive at some time point during the study. Compared with Group A cats, the lungs of Group B cats were turgid, were not collapsed, and inflated poorly with fixed pressure inflation (Fig. 1).

**Fig. 2:**
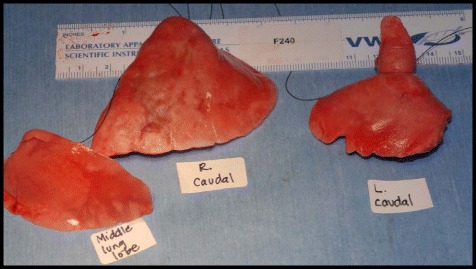
Lung lobes after fixed pressure perfusion inflation in a cat from Group B. The right caudal, right middle and portion of left caudal lung lobes after inflation with fixation solution at 14 cm H_2_O pressure. Note the uneven inflation, especially of the right caudal lobe and discoloration of the lobes. No heartworms or worm segments were identified in this cat, which was HW antibody positive

**Fig. 3:**
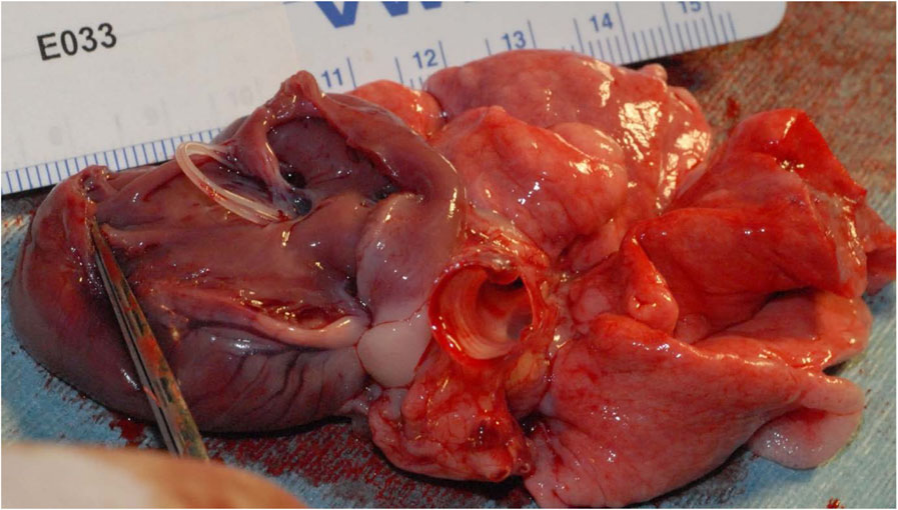
Heartworms in Group C cat after L3 *D. immitis* infection. The right ventricle is opened along the ventricular septum and reflected back. Two viable heartworms were noted in the right ventricle and extending into the pulmonary artery. The surface of the lungs was uneven and discolored. The valve leaflets were unaffected. The cat was heartworm antigen and antibody positive

All the positive control cats (Group C) were found to be infected with adult HWs upon necropsy at the end of study on Day 245 (Table 1). Live adult HWs were found in nine of 10 of the Group C cats (4.3 mean, 1–12 range) (Figs. 2 and 3). The remaining Group C cat had multiple HW fragments. All cats in Group C were HW Ab positive and eight were HW Ag positive. None of the cats had ectopic HW infection in the abdomen or the brain on serial histopathology sections.

**Table 1:** Adult heartworms at necropsy in infected untreated cats (Group C) 240 days post infection

Cat number	Viable adult worms^a^ (male, female)	Dead worm fragments^b^
E205	10 (3, 7)	2
E033	1 (1, 0)	
F150	2 (2, 0)	8
F208	12 (7, 5)	8
F204	5 (2, 3)	9
F206	1 (0, 1)	
E291	4 (1, 3)	13
E293		1 > 6 cm
G010	8 (3, 5)	11
F131		12

^a^Viability of adult worms was based on motility in warmed Hank's solution after removal

^b^Fragments are sections of dead adults and not a result of dissection during removal

#### Peripheral complete blood count

None of the cats developed any significant changes in neutrophils, monocytes, or lymphocytes during the observation period. At one of the time points (Days 70, 110, 168, or 240), peripheral eosinophilia (>1500/μL, range 1500–8100) was noted in Group A (20%), Group B (50%), and Group C (100%) (Table 2). At one of the time points (Day 70, 110, 168, or 240), peripheral basophilia (>100/μL, range 100–294) was noted in noted in cats in Group A (50%), Group B (40%), and Group C (60%) but was not consistent for individual cats over time. Basophilia occasionally was noted in the absence of eosinophilia (>1500/μL) in Groups B and C at various days PI.

**Table 2:** Number of cats with peripheral eosinophilia (>1500/μL) after L3 *D. immitis* infection

Treatment Group^a^	Day 0		Day 70		Day 110		Day 168		Day 240	
	> 1500	Range	> 1500	Range	> 1500	Range	> 1500	Range	> 1500	Range
Group A	0	421–1125	1	50–1800	1	287–1550	0	150–930	0	145–822
Group B	0	295–1500	2	293–2205	5	325–6100	3	183–8219	2	450–5280
Group C	1	512–2210	7	449–5067	7	0–7282	9	558–8761	8	800–9864

^a^Treatment groups: Group A (10), selamectin topical; Group B (9), oral ivermectin initiated Day 70 PI, Group C (10), infected, untreated

### Bronchoalveolar lavage

Cats in both Groups B and C had a significantly (*p* < 0.05) higher percentage of eosinophils on BAL on Days 110 and 168 compared with Group A cats (Table 3). The presence of increased BAL eosinophilic cytology was not consistent across time points in individual cats.

**Table 3:** Number of cats per group^a^ with bronchoalveolar lavage eosinophilic cytology after L3 *D. immitis* infection

	Day 0			Day 110			Day 168			Day 240		
Grade^b^	Group A	Group B	Group C	Group A	Group B	Group C	Group A	Group B	Group C	Group A	Group B	Group C
0	10	8	9	8	4	1	9	5	3	9	4	4
1	0	1	1	0	1	1	0	1	1	1	1	3
2	0	0	0	2	3	3	1	1	3	0	1	1
3	0	0	0	0	1	4	0	1	2	0	2	1

^a^Treatment groups: Group A, selamectin topical; Group B, oral ivermectin initiated on Day 70 PI; Group C, infected, untreated

^b^Eosinophils as percent of nucleated cells on BAL by rank: Grade 0 < 16%, Grade 1 = 17–35%, Grade 2 = 36–60%, Grade 3 > 61%

An elevated peripheral eosinophilia (>1500/μL) and a cytologic BAL eosinophilia response (eosinophils >16%) did not demonstrate a significant correlation to each other (Pearson’s correlation, *r* = 0.389) within groups or within data from all groups combined. In individual cats, increased eosinophilia (>16%) on BAL cytology was frequently noted with normal peripheral eosinophil counts. Elevated eosinophilic BAL cytology (>60%) was recorded in cats with modest eosinophilia (<2500/μL), and elevated peripheral eosinophilia (>3000/μL) was associated with normal BAL cytology.

### Serology

Eight of the Group A cats were HW Ab positive on at least one sampling interval. None of the Group A cats were HW Ag positive.

**Fig. 4:**
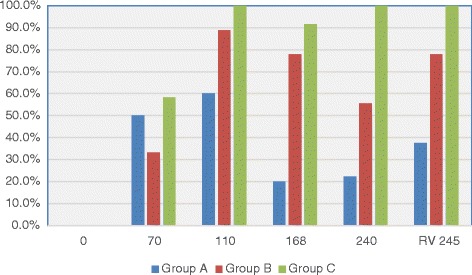
Percentage of cats heartworm antibody positive in each group at days after L3 *D. immitis* infection*.* Treatment groups: Group A, selamectin monthly initiated on Day 28; Group B, oral ivermectin every 2 weeks initiated on Day 70 PI; Group C, infected, untreated. Days after infection listed on bottom axis and RV 245 is sample collected from right ventricle immediately post mortem

**Fig. 5:**
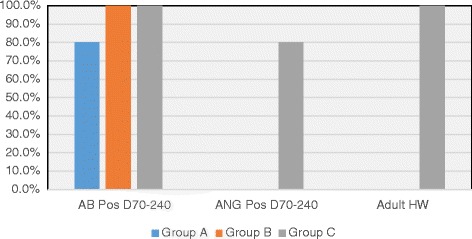
Serology results in cats after *D. immitis* infection. Treatment groups: Group A, selamectin monthly initiated on Day 28; Group B, oral ivermectin every 2 weeks initiated on Day 70 PI; Group C, infected, untreated. Percentage of cats in each group that were positive at any time point for heartworm antibody (AB Pos Days 70–240) or antigen (ANG Pos Days 70–240). Percentage of cats that had heartworms or worm fragments at necropsy (Adult HW)

At one of the data points of collection (Days 70, 110, 168, or 240), HW Ab titers were positive in all cats in Groups B and C, and 80% of cats in Group A. The highest optical density (OD) for antibody titer varied over time for each cat (OD >0.4 positive, range 0.40–5.00, with 5.00 as maximum of assay) but by 8 months PI for Group B cats (abbreviated infection), 50% of the cats were negative (< 0.4 OD) (Fig. 4). The only positive antigen results were in Group C cats on Day 168 (33% of cats, OD > 0.05, positive range 0.386–0.546) and Day 240 (80% of cats, OD range positive 0.204–0.613) (Fig. 5). All Ag-positive cats had a viable female HW at necropsy except for one cat with a dead female HW (Table 1, E293).

### Radiographs

**Fig. 6:**
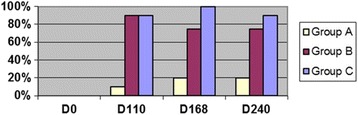
Percentage of cats with radiographic bronchial-interstitial scores of ≥1 (Score 0–3) after L3 *D. immitis* infection*.* Percentage of cats in each group with score ≥ 1 (Score 0–3). Treatment groups: Group A, selamectin monthly initiated on Day 28; Group B, oral ivermectin every 2 weeks initiated on Day 70 PI; Group C, infected, untreated. Days after infection listed on bottom axis

**Fig. 7:**
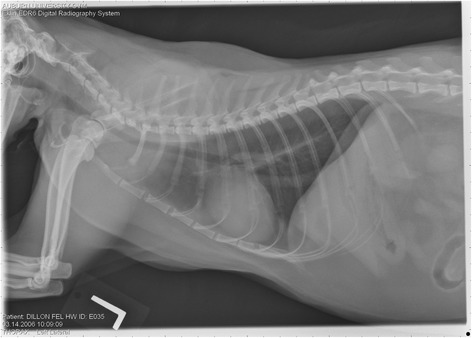
Group A (infected, selamectin monthly initiated on Day 28) cat. Lateral Radiograph on Day 240. Normal lateral thoracic radiographs in a cat from Group A (selamectin monthly initiated Day 28 PI) on Day 240 after L3 *D. immitis* infection. There was no significant change in radiographs in any cat from Day 0, 110, 175, or 240. All Day 0 radiographs were normal for all cats

On Day 240, cats in Groups B and C had radiographic scores for main pulmonary artery, arterioles, and bronchial interstitial changes which were not consistently different from each other (Figs. 6, 7 **,** and 8) but both were significantly different (*p* > 0.05) from Group A. None of the cats in Group A had abnormal arterial scores on any day evaluated. On Day 110, cats in both Groups B and C had abnormal radiographs (Figs. 9, 10, 11, 12, 13, 14, 15, 16, 17, 18, 19, 20) and cats in Groups B and C could not be distinguished from each other on Days 110, 168, and 240. Pulmonary arterial scores of ≥2 developed in Group B (20%) and Group C (40%) cats. Bronchial-interstitial scores of ≥2 developed in Group B (50%) and Group C (60%) cats.

**Fig. 8:**
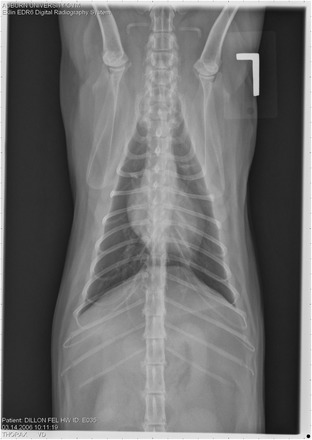
Group A cat (infected, selamectin monthly initiated on Day 28). Ventrodorsal radiograph on Day 240. Normal lateral thoracic radiographs in a Group A cat (selamectin monthly initiated on Day 28 PI) on Day 240 after L3 *D. immitis* infection. There was no significant change in radiographs in any cat from Day 0, 110, 175, or 240. All Day 0 radiographs were normal for all cats

**Fig. 9:**
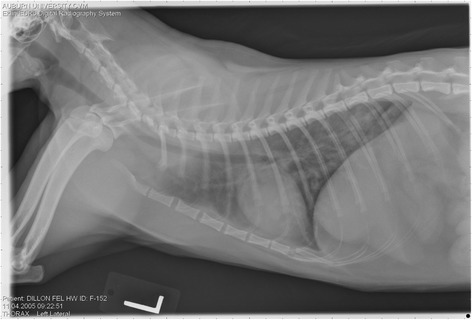
Radiographs of a cat from Group B: Day 110. Left lateral and ventrodorsal radiographs of a cat from Group B (oral ivermectin every 2 weeks initiated on Day 70 PI) with no heartworms at necropsy on Day 240. This cat was heartworm antibody and antigen negative. Day 0 radiographs were normal, and did not differ from Group A cats on Day 0–240 and Group C cats on Day 0. The lungs revealed an interstitial and bronchial pattern on Day 110 more marked in the caudal lung lobes which became more severe on Day 175, with anterior lobes becoming more involved. The pulmonary arteries could not be clearly defined because of the severity of the lung parenchymal disease. On Day 240, the lung disease continued to be evident and the left caudal pulmonary artery was slightly more pronounced beyond the cardiac silhouette. The heart and main pulmonary artery were not enlarged

**Fig. 10:**
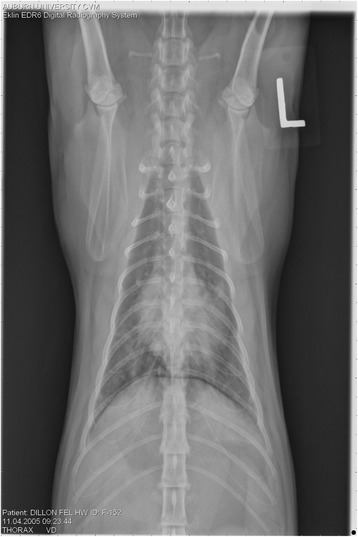
Radiographs of a cat from Group B: Day 110. (See Fig. 9 caption for details)

**Fig. 11:**
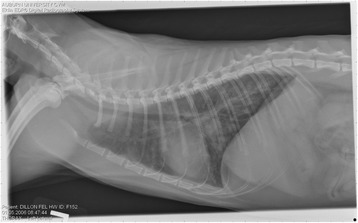
Radiographs of a cat from Group B: Day 175. (See Fig. 9 caption for details)

**Fig. 12:**
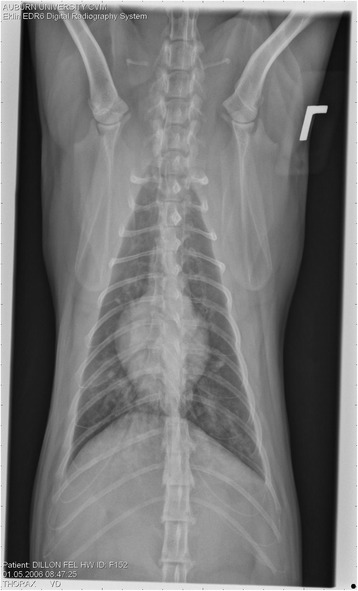
Radiographs of a cat from Group B: Day 175. (See Fig. 9 caption for details)

**Fig. 13:**
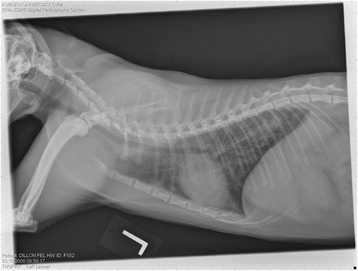
Radiographs of a cat from Group B: Day 240. (See Fig. 9 caption for details)

**Fig. 14:**
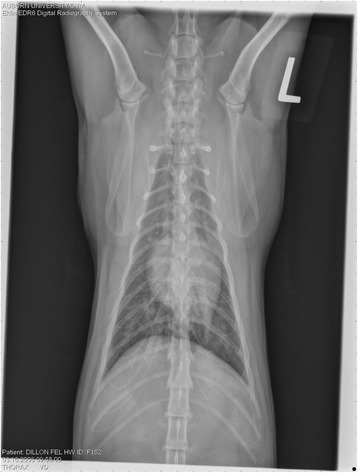
Radiographs of a cat from Group B: Day 240. (See Fig. 9 caption for details)

**Fig. 15:**
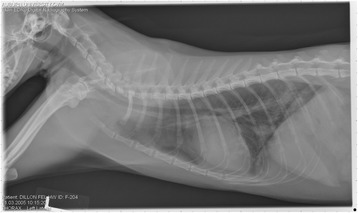
Radiographs of a cat from Group C: Day 110. Left lateral and ventrodorsal radiographs of a cat from Group C (infected no treatment, with two heartworms at necropsy) that was heartworm antibody and antigen positive. Day 0 radiographs were normal, and did not differ from Group A cats on Days 0–240, and Group B on Day 0. The lungs revealed an interstitial and bronchial pattern on Day 110, more marked in the caudal lung lobes, which became more severe on Day 175 with anterior lobes becoming more involved. The pulmonary arteries could not be clearly defined because of the severity of the lung parenchymal disease. On Day 240, the lung disease continued to be evident and the left caudal pulmonary artery was slightly more pronounced beyond the cardiac silhouette. The heart and main pulmonary artery was not enlarged

**Fig. 16:**
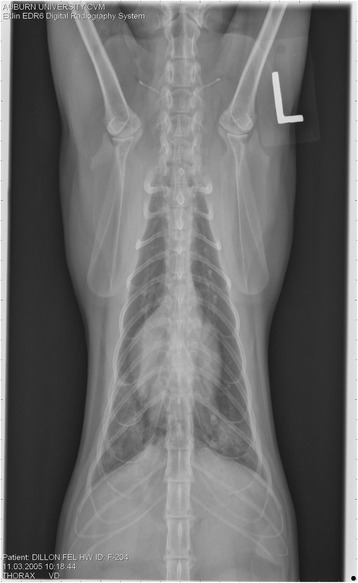
Radiographs of a cat from Group C: Day 110. (See Fig. 15 caption for details)

**Fig. 17:**
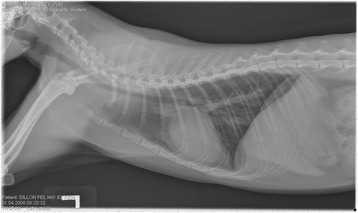
Radiographs of a cat from Group C: Day 175. (See Fig. 15 caption for details)

**Fig. 18:**
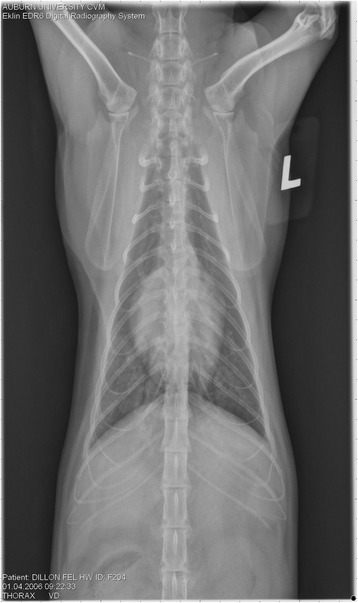
Radiographs of a cat from Group C: Day 175. (See Fig. 15 caption for details)

**Fig. 19:**
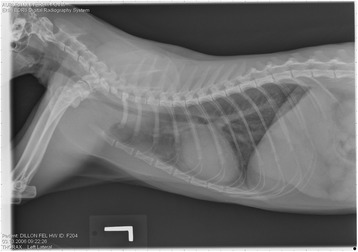
Radiographs of a cat from Group C: Day 240. (See Fig. 15 caption for details)

**Fig. 20:**
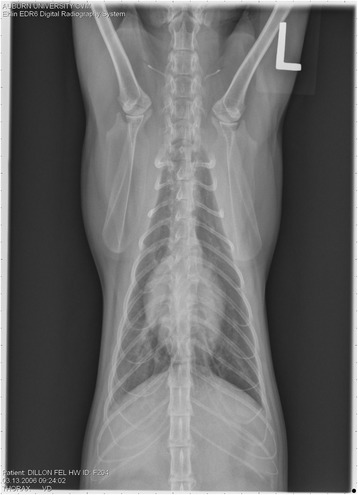
Radiographs of a cat from Group C: Day 240. (See Fig. 15 caption for details)

### Lung pathology

**Fig. 21:**
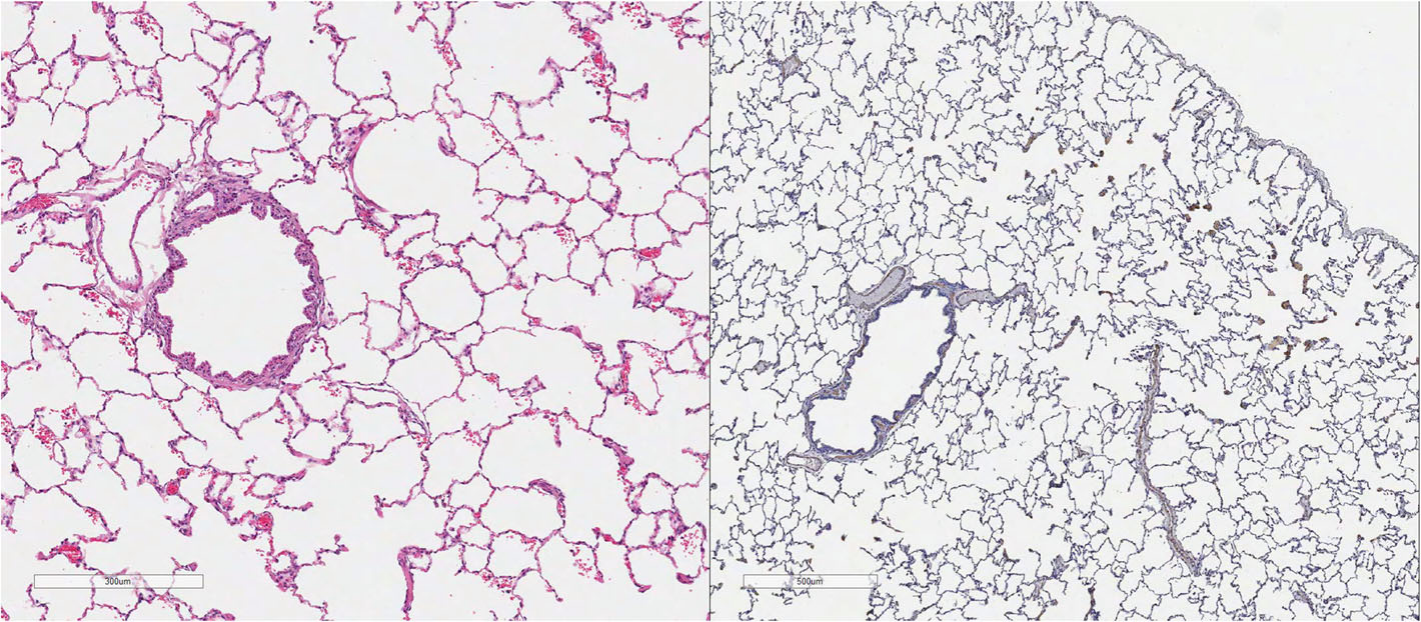
**a** (*left*) and **b** (*right*). . Histopathology of feline lung with H & E stain or alpha-smooth muscle actin stain after L3 *D. immitis* infection. The cats in Group A were generally normal. **a** (*left)*, **b** (*right*)] with occasional isolated area of infiltration of smooth muscle [Fig. 22 **a** (*left*), B (*right*)], especially in small arterioles. Cats in Group B had severe inflammation around airways [Fig. 23 **a** (*left*), **b** (*right*)] and interstitial and pulmonary arterial proliferation of smooth muscle (Figs. 22–24) not directly associated with bronchioles or arterioles. The pulmonary arterial smooth muscle proliferation was consistent with heartworm disease. Although all cats in Group A did not consistently have lung pathology (Fig. 25 **a**, *left*), in some lobes isolated areas of myofibrocyte proliferation were observed (Fig. 25 **b**, *right*). Cats in Group C had disease similar to Group B cats except that the endothelial proliferation in pulmonary arteries (Figs. 26, 27) was noted in isolated major vessels and the airway and interstitial disease was more consistent that observed in Group B cats

**Fig. 22:**
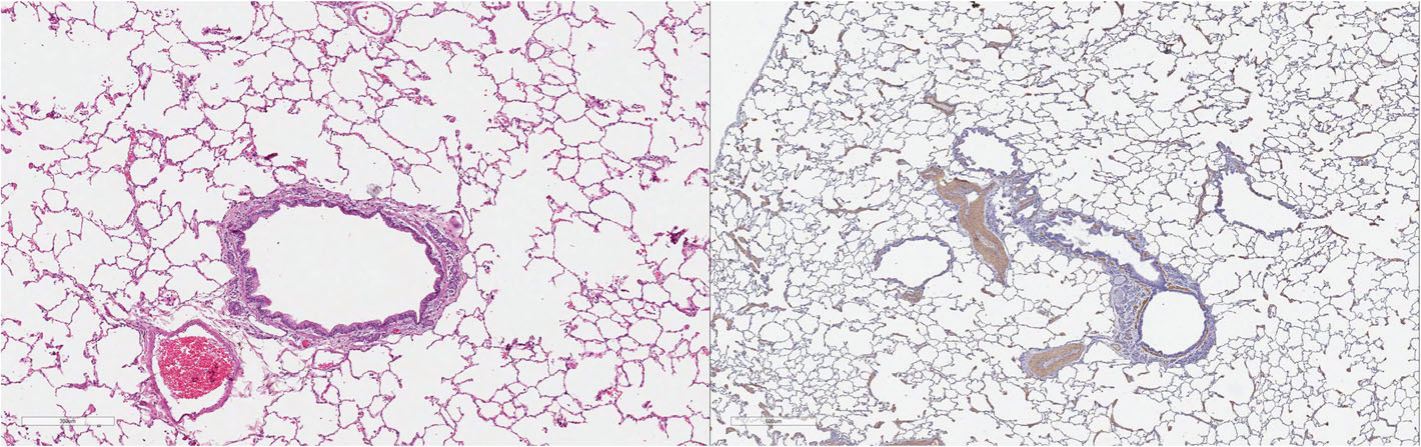
**a** (*left*) and **b** (*right*). Histopathology of feline lung with H & E stain or alpha-smooth muscle actin stain after L3 *D. immitis* infection. (See Fig. 21 caption for details)

**Fig. 23:**
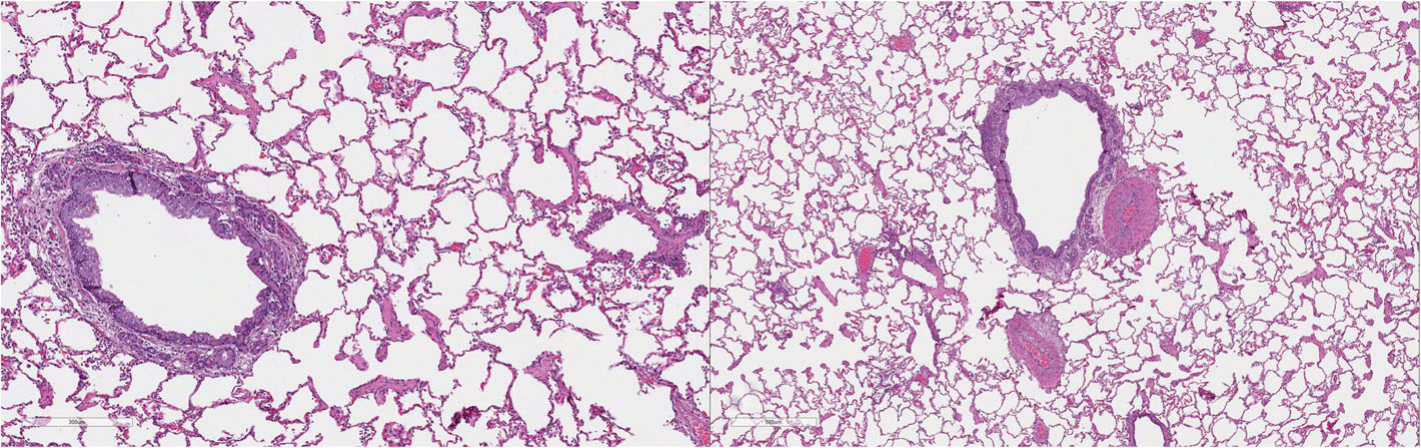
**a** (*left*) and **b** (*right*). Histopathology of feline lung with H & E stain or alpha-smooth muscle actin stain after L3 *D. immitis* infection. (See Fig. 21 caption for details)

**Fig. 24:**
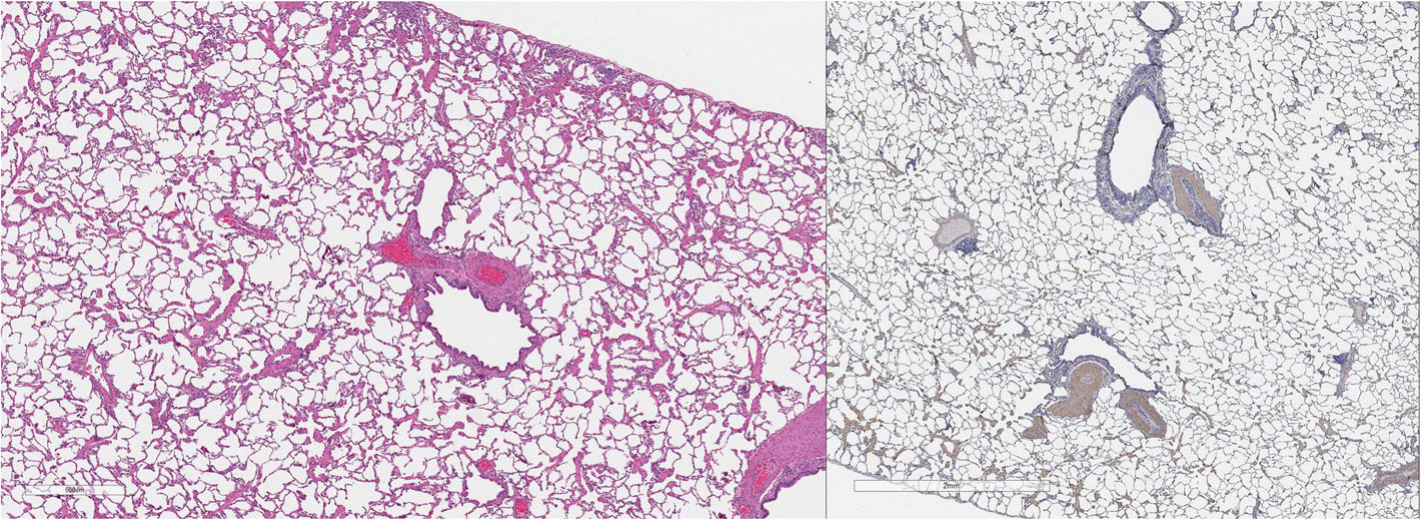
**a** (*left*) and **b** (*right*). Histopathology of feline lung with H & E stain or alpha-smooth muscle actin stain after L3 *D. immitis* infection. (See Fig. 21 caption for details)

**Fig. 25:**
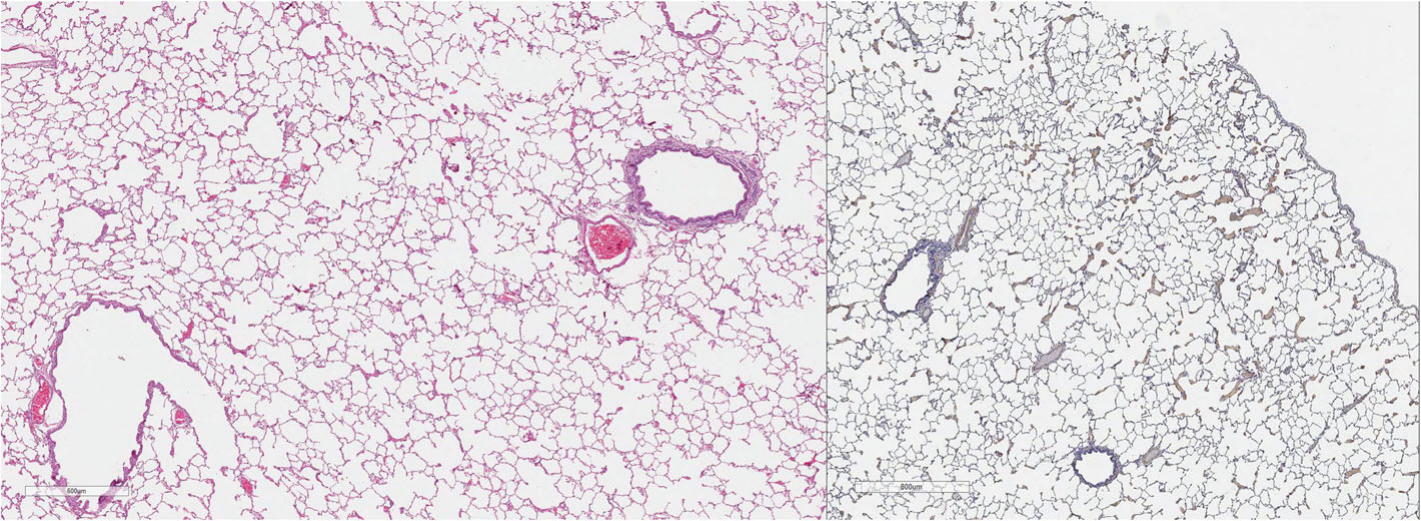
**a** (*left*) and **b** (*right*). Histopathology of feline lung with H & E stain or alpha-smooth muscle actin stain after L3 *D. immitis* infection. (See Fig. 21 caption for details)

**Fig. 26:**
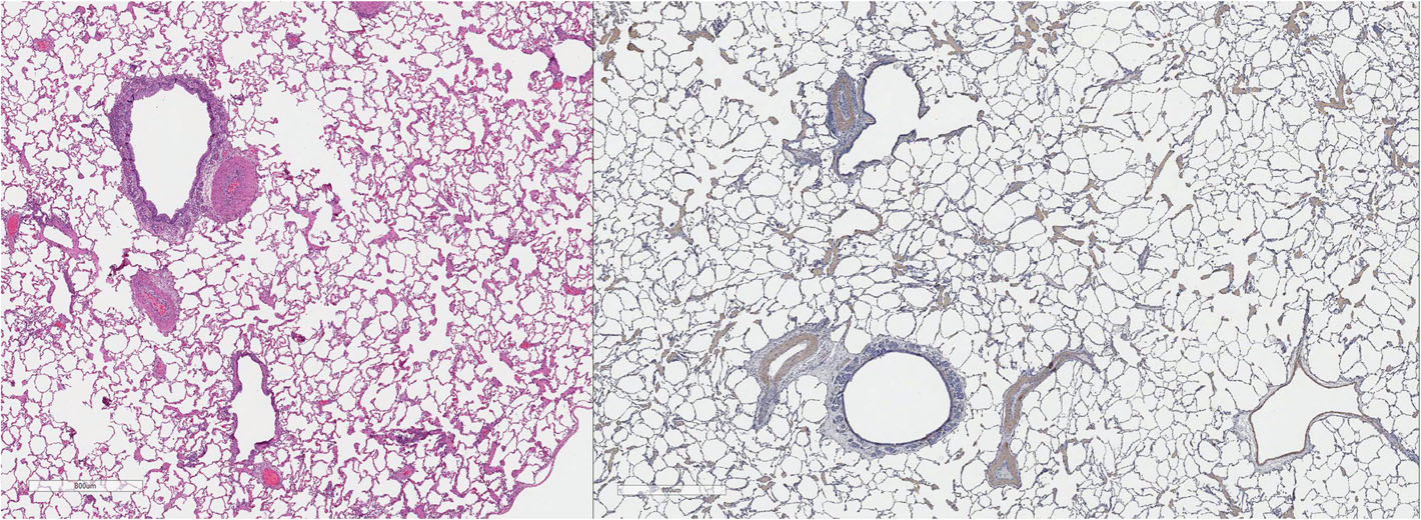
**a** (*left*) and **b** (*right*). Histopathology of feline lung with H & E stain or alpha-smooth muscle actin stain after L3 *D. immitis* infection. (See Fig. 21 caption for details)

**Fig. 27:**
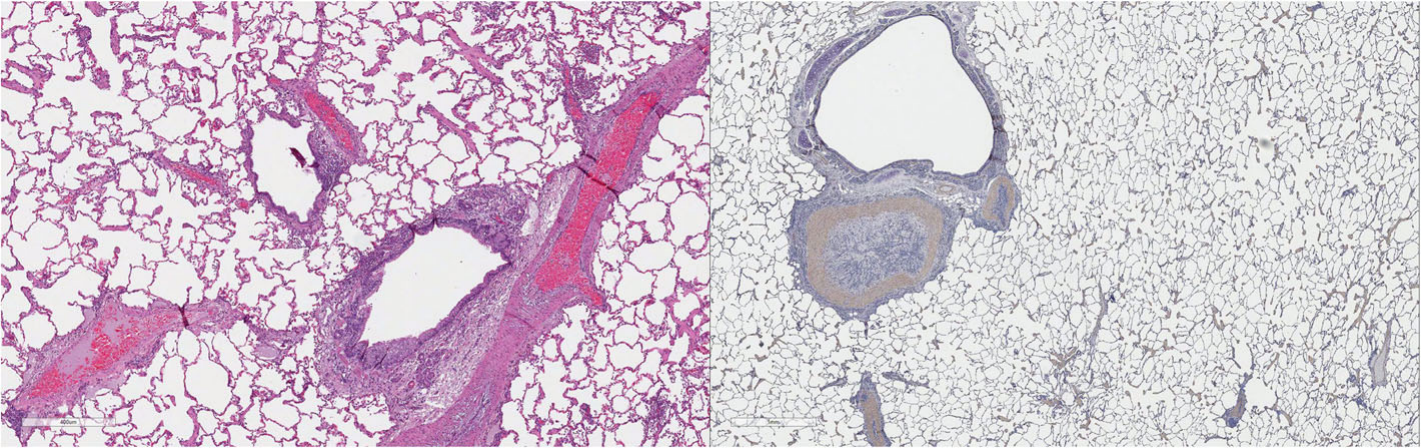
**a** (*left*) and **b** (*right*). Histopathology of feline lung with H & E stain or alpha-smooth muscle actin stain after L3 *D. immitis* infection. (See Fig. 21 caption for details)

Cats in both Groups B and C had abnormal lungs with turgidity on gross examination (Figs. 2, 3). Lungs in Group A were considered normal on gross examination. Evaluation of lung scores (0–3) demonstrated a statically significant difference between cats in Groups B and C versus Group A for subjective scoring and for morphometric lumen to wall ratio (Table 4, Figs. 21, 22, 23, 24, 25, 26, 27). Significant increases in smooth muscle actin (SMA)-positive tissue were associated with peribronchial and interstitial changes. Isolated localized areas of smooth muscle proliferation in airway and interstitium were noted in some cats in Group A (Fig. 21). As a result, the subjective pulmonary parenchymal lung scores (range grade 0–3) in Group A were not considered normal and the difference between Groups A and B was not significantly different because of this variability in lesion distribution in individual cats.

**Table 4:** Histopathologic grading (scoring 0–3) and lumen to wall ratio of fixed perfused in cat lung

Site	Group^a^	Lesion Score	Significance^b^
Bronchus	A	1.40	
	B	1.89	0.010
	C	2.40	0.003
Bronchiole	A	1.65	
	B	1.89	
	C	2.50	0.009
Alveolus/Smooth Muscle	A	1.40	
	B	2.11	0.050
	C	2.60	0.001
Arteriole	A	1.75	
	B	2.39	0.002
	C	2.70	0.002
Pulmonary Artery	A	1.20	
	B	2.22	0.001
	C	2.60	0.001
Bronchiole Lumen to Wall Ratio^c^	A	1.416	
	B	1.016	0.001
	C	1.080	0.001
Pulmonary Arteriole Lumen to Wall ratio^c^	A	0.332	
	B	0.528	
	C	1.187	0.001

^a^Treatment groups: Group A, selamectin topical; Group B, oral ivermectin initiated on Day 70 PI; Group C, infected, no treatment

^b^Significance difference (ANOVA p > 0.05) noted between groups for each variable examined, and pairwise comparison (Holm-Sidak) significance in table notates differences between group B or C compared to group A

^c^Lumen to wall ratio is morphometric measure of area of lumen compared to area of wall of at least 5 bronchioles and 5 arterioles from each cat. Group B was not significantly different from Group C for any variable

In morphometric measure of bronchioles, lumen to wall thickness of the airway, and wall thickness, Groups B and C were not different from each other but both groups were significantly different than Group A (Table 4). The arteriole lumen to wall thickness demonstrated that Group C cats had significant thickening of the wall compared with Group A and Group B, but the severity of the lesions was inconsistent within the cats in Group B, and was not significantly different from Group A. Random distribution of interstitial SMA positive fibrocytes was noted in cats in Groups B and C and was confirmed on examination of lung sections in other areas of the lobe.

#### General necropsy results

Data from histopathology of other organs are not included in this paper. Gross serial sections of the brain did not suggest the presence of aberrant sites of HWs.

## Discussion

In this experimental model, the HW life cycle was abbreviated with oral doses of ivermectin initiated at 70 days PI demonstrated by the absence of adult HWs or worm fragments at Day 245 PI. In Group B, however, the early stage of immature adult HW that arrive in the pulmonary vasculature at 70 to 90 days PI, which did not develop into adults, did induce severe pulmonary airway, interstitial, and arterial lung lesions. The clinical implications of Heartworm-Associated Respiratory Disease (HARD) as a consequence of a single unsuccessful infection suggest a broader definition of HW disease in the cat. Based on CBC, BAL, serologic antibody testing, and radiographic evaluation on Days 110, 168, and 240, differences could not be discerned in cats from Group B (no HWs or fragments at necropsy) and Group C (live HWs at necropsy). The bronchial and interstitial lung disease was evidenced by thoracic radiographs on Day 110 PI in both Groups B and C. Radiographs on Days 175 and 240 also showed parenchymal lung and pulmonary arterial changes in Groups B and C. The selamectin (Revolution®)-treated cats (Group A, monthly dosing starting on Day 28 PI) did not develop radiographically evident changes, although an eosinophilic BAL cytology was noted in some cats. The positive HW antibody that developed in Group A cats suggest that the L3 did live and develop long enough to initiate an immune response typically observed by 2 to 3 months PI. The concerns over the interstitial lung changes noted in some Group A cats are addressed in a separate study [18].

Often the pulmonary arterial (PA) enlargement on VD radiographs could not be visualized because of the lung changes (Figs. 9, 10, 11, 12, 13, 14, 15, 16, 17, 18, 19, 20). The more prominent pulmonary artery, however, was more frequently noted and scores were higher (>1) in the Group C cats as compared with the Group B cats. Because some cats with adult HWs had normal PA segments and some abbreviated infections had enlarged PA, this characteristic is not a clinical tool which can be used with certitude. No evidence of enlargement of the cardiac silhouette or a prominent PA bulge, as in canine HW disease, was noted which is typical of feline HW disease. Four of the Group C cats with normal PA radiographic scores on Day 240 had live HWs at necropsy on Day 245. The inability to distinguish consistent differences in radiographic parenchymal lung pattern between Groups B and C reveals the limitation in radiographs to define active adult infections from HARD or previous infections. The clinical implications are that radiographs, even combined with antibody serology, cannot distinguish between a current adult worm infection and lung disease associated with previous unsuccessful immature adult infections (HARD).

The histologic scores of lung parenchyma in Group B cats (HARD) included typical lesions of adult HW infection in the pulmonary arteries, arterioles, bronchi, bronchioles, and alveolar interstitial areas. The bronchial disease was marked and resulted in increased wall thickness as noted on subjective scoring and the measured increased wall to lumen ratio. The contributions of peribronchial inflammation and interstitial reaction were more severe than an increase in smooth muscle bronchial wall thickness in Groups B and C. The interstitial proliferation of smooth muscle was a marked characteristic often not directly associated with the airway or arterial system. Smooth muscle actin stain demonstrated a significant increase in pulmonary arterial and arteriolar smooth muscle but often in random arteries even in the same lung lobe section. The radiographic scoring of increased bronchial lesions was most commonly associated with histologic peribronchial and interstitial lung reaction rather than with bronchial epithelial changes. In Group A cats, the subtle changes in the interstitium, pulmonary arterioles, and pulmonary arteries in isolated cats in the group were considered abnormal and may reflect pulmonary reaction to developing stages of HWs not related to adult HWs. Further complicating the clinical implication is that cats which were treated with selamectin initiated 28 days after a single HW infection did not develop adult worms and were radiographically normal, but had random histologic lung changes and developed positive antibody titers for 2 to 8 months after the infection. These concerns of pre-cardiac tissue stages of HW larvae are addressed in a separate study [18].

Despite proliferation of smooth muscle in the airways, in vitro bronchial wall reactivity in HW-infected cats has been demonstrated to be blunted and the airways are nonresponsive to dilation or constriction [19]. Evidence of a hyper response, such as “asthma like” bronchial constriction, could not be demonstrated despite the histologic evidence of smooth muscle proliferation and eosinophilic cytology on BAL examination. The lack of significant increases in smooth muscle in the walls of the airways is consistent with these findings. The similar histologic pulmonary lesions noted in both Group B and C cats suggest that HARD and adult HW infections do not have an exaggerated bronchial constrictive response but rather have chronic bronchial disease and restrictive lung disease.

The diffuse bronchial lung disease and diffuse smooth muscle interstitial component would suggest that HARD and adult HW infections induce a restrictive lung disease similar to that described in *Toxocara cati* infections. The bronchial disease from HARD is more severe that the airway disease in *T. cati* infections [20]. The elevated percentage of eosinophils on BAL cytology was not predictive of the severity of the bronchial lesions histologically or radiographically in the current study and is consistent with blunted bronchial ring reactivity in cats with adult HWs [19]. Similar patterns of high eosinophil counts on BAL cytology and pulmonary radiographic lesions have been demonstrated in *T. cati*–infected cats where in vitro bronchial ring reactivity was normal [20]. Clinically, in lung disease of HARD and *T cati* infection, the BAL eosinophilic cytology would not be predictive of airway disease or bronchial ring reactivity.

**Fig. 28:**
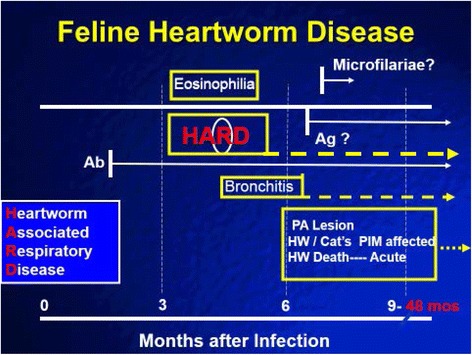
Diagram of feline heartworm disease and HARD. Heartworm-associated respiratory disease (HARD) is induced at the first arrival of immature adult worms as early as 70 to 90 days after infection. The inflammation develops even if the cat “self-cures” and all immature adults die, and no adult heartworms develop. The lung lesions continue for up to 8 months after infection and perhaps longer. The antibody (Ab) response is present if cats are started on selamectin 28 days after the infection even if immature adults do not reach the heart. The antibody response continues after the arrival of immature adults; in cats which develop only the immature adults with HARD, the antibody response is present in 50% of the cats 8 months after the infection. Cats that develop the adult heartworms continue to live for up to 4 years, often with no symptoms, and the cats develop a decreased responsiveness of their pulmonary intravascular macrophages (PIM). When adult heartworms eventually die, acute symptoms may develop

The development of HARD as a consequence of only immature adult HWs was associated with an eosinophilic BAL, radiographic changes, and a positive antibody titer at Day 110 PI (Fig. 28). The positive antibody titer attenuated over time and became normal in 50% of Group B cats by Day 240 PI. The radiographic lung disease and BAL changes continued relatively unchanged, however, on Days 168 and 240. Because in HARD the HWs do not develop into adults, the evidence of infection by HW serologic testing may be negative just months after the infection even with continued radiographic lesions. In a regional clinical study of 210 client-owned cats, [16, 17] coughing cats presented with radiographic lesions of bronchial/interstitial disease that were HW Ab positive but over the next 3 months some of these became HW antibody negative. This has been described as a “self-cure” [11, 13, 14]. In cats in the clinical study, however, the majority continued to have abnormal radiographs at the 3-month recheck, with approximately one third with improvement while 25% became more severe [15]. This group of cats was typically treated with corticosteroids and improved clinically, and normally would not have had recheck serology and radiographs. The concept of HARD associated with only immature adult infections is also supported by a study of random-sourced cats in which cats were reported with pulmonary artery lesions consistent with HW infection and were serologically positive but had no evidence of adult HWs at necropsy [21, 22] (Fig. 28).

The severity of the inflammatory response by Day 110 in Group C cats, in which adult HWs developed, is consistent with a naturally higher mortality in cats of immature adults that arrive in the heart and lungs as compared with dogs, and is consistent with the authors’ (ARD, BB) personal experience in feline HW studies. No published studies are available as to the number of immature adults present in a cat by Day 90 PI. The challenge to these cats was large (100 L3) and is a proof of the concept study of HARD, which is consistent with clinical studies. Further, in endemic areas cats would be expected to have multiple infections per mosquito season and be reinfected each year. The clinical outcome of repeated small infections and potential for heightened immune responses warrant further investigation.

HARD is compatible with the clinical presentation of a cat with radiographic bronchial-interstitial lesions with a positive antibody titer which over time seroconverts to negative. Immature adults create disease and induce an inflammatory BAL cytology with a positive HW Ab titer, but are unsuccessful in developing into an adult HW. If a cat is examined after the initial insult of HARD, the antibody titer could be negative yet the lung disease could remain for an undermined period of time.

## Conclusion

The arrival of immature adult HWs by 110 days after infection is associated with an intense airway and lung parenchymal reaction which can be noted on clinical diagnostic evaluation by BAL, CBC, serology, and thoracic radiography. Immature adult HWs which did not develop into adult HWs can induce HARD that is histologically and radiographically evident 8 months after the infection and the antibody titer was negative in 50% of cats 8 months PI. Selamectin (Revolution®) administered starting 1 month after infection prevented the development of immature adult and mature adult HWs but 80% of cats developed a positive Ab titer that was still positive in 20% of cats 8 months after the infection.

## Abbreviations

AAALAC: Association for assessment and accreditation of laboratory animal care
Ab: Antibody
Ag: Antigen
BAL: Bronchoalveolar lavage
CBC: Complete blood count
ELISA: Enzyme-linked immunosorbent assay
HARD: Heartworm-associated respiratory disease
HW: Heartworm
IM: Intramuscular
IP: Intraperitoneal
IV: Intravenous
PI: Post infection
SMA: Smooth muscle actin
SQ: Subcutaneous
VD: Ventrodorsal
